# HEC-ASD: a hybrid ensemble-based classification model for predicting autism spectrum disorder disease genes

**DOI:** 10.1186/s12859-022-05099-7

**Published:** 2022-12-21

**Authors:** Eman Ismail, Walaa Gad, Mohamed Hashem

**Affiliations:** Information Systems Department, Faculty of Computer and Information Sciences, Ain Shams University, Cairo, Egypt

**Keywords:** Gene prediction, Boosting techniques, Gene ontology, Ensemble learning, Functional gene network, Gene classification

## Abstract

**Purpose:**

Autism spectrum disorder (ASD) is the most prevalent disease today. The causes of its infection may be attributed to genetic causes by 80% and environmental causes by 20%. In spite of this, the majority of the current research is concerned with environmental causes, and the least proportion with the genetic causes of the disease. Autism is a complex disease, which makes it difficult to identify the genes that cause the disease.

**Methods:**

Hybrid ensemble-based classification (HEC-ASD) model for predicting ASD genes using gradient boosting machines is proposed. The proposed model utilizes gene ontology (GO) to construct a gene functional similarity matrix using hybrid gene similarity (HGS) method. HGS measures the semantic similarity between genes effectively. It combines the graph-based method, such as Wang method with the number of directed children’s nodes of gene term from GO. Moreover, an ensemble gradient boosting classifier is adapted to enhance the prediction of genes forming a robust classification model.

**Results:**

The proposed model is evaluated using the Simons Foundation Autism Research Initiative (SFARI) gene database. The experimental results are promising as they improve the classification performance for predicting ASD genes. The results are compared with other approaches that used gene regulatory network (GRN), protein to protein interaction network (PPI), or GO. The HEC-ASD model reaches the highest prediction accuracy of 0.88% using ensemble learning classifiers.

**Conclusion:**

The proposed model demonstrates that ensemble learning technique using gradient boosting is effective in predicting autism spectrum disorder genes. Moreover, the HEC-ASD model utilized GO rather than using PPI network and GRN.

## Introduction

The prevalence of autism among children is one of the most important topics that must pay attention to know the causes of the disease and to take the initiative to find solutions for effective treatment. Autism is a disorder that belongs to a group of developmental disorders called autism spectrum disorders (ASD) [1], which appears in childhood, often before the age of three. Although the symptoms of autism vary from one patient to another, all ASDs affect a child’s ability to communicate with other people around him and develop relationships with them. Many researchers have focused on the search for autism through the symptoms that appear on the patient, but few are interested in finding the genetic causes of the disease, so in this research, we are interested in predicting genes of autism.

Most studies use machine learning (ML) to predict the genes of autism spectrum disorder, which defines the problem as binary classification, positive (disease genes) and negative (non disease genes). Many genes were produced through extensive research, so identifying disease-causing genes from this comprehensive database has become difficult [1]. However, machine learning can analyze such large amount of data and identify the most distinct traits, which helps to predict disease-causing genes. Machine learning techniques are helpful in different biomedical problems such as drug discovery [2, 3], gene prediction [4, 5], disease gene interactions [6], genome annotations [7], gene regulatory network derivation [8], microarray data classification [9],and protein function prediction [10]. Some predictive models [11] were proposed to identify genes related to ASD and gene sets associated with autism according to specific cell types. They give higher priority to high-confidence candidates genes from Simons Foundation Autism Research Initiative (SFARI) to construct a predictive model. Their predictive model is multi-labeled to predict the type of cell associated with genes and the set of candidate genes that may be used as an identifier to diagnose ASD.

A support vector machine model was built in [12] to identify ASD risk genes and their influence on the temporospatial areas in the brain at different times using gene expression. Some researchers utilized deep learning techniques in gene prediction models [13], the DeepHE model was proposed to train a multilayer network using DNA sequence data and the data from the protein-to-protein network (PPI). These models that depend only on one PPI network gained low performance, as the PPI network is not fully completed till now (do not include whole protein interactions) and has more noise data connection.

Moreover, a weighted classifier using support vector machine(SVM) was proposed to detect the association relationships between genes in the brain and ASD [14]. They utilized a hybrid network to train a weighted SVM classifier. This network combined the PPI network, gene expression (GE), and brain network genes, then evaluated on the highest confidence genes from SFARI dataset. This model had some restrictions as the PPI network and gene expression did not have representation for weak connections, which limit the classifier performance.

Recent studies utilized gene ontology(GO) [15, 16] to predict disease genes, as it is believed that two genes are similar if their phenotypes are similar. In [17], a group of genes may belong to the same biological process (the same branch in GO) if these genes are disrupted by the same genetic variants. Prediction of gene function [18] using gene ontology may be categorized into four categories:

- Prediction using internal relationships between gene ontology terms.
- Prediction with dimension reduction for gene ontology terms matrix.
- Prediction using different species of gene data.
- Prediction using semantic similarity between genes.

Gene ontology (GO) is constructed as a hierarchical-directed acyclic graph of gene ontology terms and the relations between them. In the first category, prediction using internal relations between terms can divided into two types: the trivial relationships between terms, including the occurrence of the terms, and the second type uses their hierarchical relationships to measure the similarity. In [19], they predict new terms to annotate genes using the term occurrence of the same two genes. ProDM [20] is a proposed algorithm that uses the maximum dependencies between genes features and genes annotations using GO to predict new annotation terms for genes. In [21], the Noisy GO annotation model was proposed to predict disease genes using the taxonomic relationships of GO terms using its hierarchical graph and measuring the semantic similarity between genes using their annotation terms.

The second category, dimension reduction for gene ontology term matrix, can be done using two different techniques; applying matrix factorization [22, 23], which reduces the matrix of genes terms to predict genes’ new annotations, or using a hashing function. Ref. [24], clusterDCA method is proposed to perform matrix factorization on gene ontology terms. Their method uses the singular value decomposition (SVD) technique into two adjacent matrices obtained from GO DAG. It reduces the noise in the two matrices producing low matrix dimensions that infer the associative relationships between genes and their annotation terms. NoisyGO model does not check reliability as it does not remove the noisy annotations in measuring the semantic similarity between genes. Moreover, NOGOA model is proposed in [25] gives weight to GO annotations to distinguish between genes and detect noises using a weight of genes evidence codes.

Furthermore, using hashing solutions is effective in speeding up the process of measuring the semantic similarity between genes [26], researchers build hashing functions for coding gene ontology terms to compress vast GO terms, [26] build a network that includes GO terms with their binary code, then calculating the semantic similarity between genes utilizing hamming distance function to predict genes. In the third category [27], they build a new network that combines GO hierarchical structure and PPI network and one or more species of sequenced data homology to improve the prediction of gene function.

The last prediction category uses semantic similarities between genes choosing adjacent genes and using their annotation to measure the similarity. Measuring the semantic similarity between genes using GO is divided into two types based on the taxonomy of GO terms. The first type is a pairwise method, which uses two combination measures, maximum strategy [28] and best match average strategy (BMA), which is a combination of max and average [29]. The second type is groupwise, which traits terms as vectors or subgraphs from DAG to measure the semantic similarity [30], SORA method uses the information content (IC) for each term to make an overlap ratio and measure the similarity between their associated genes. Moreover, [31] they use GO to measure the semantic similarity between genes contributed to the same biological process trained on the ASD SFARI dataset, and evaluated using stratified cross-fold validation using different classifiers.

Deep learning (DL) algorithms are used to predict genes associated with a specific disease, but some studies showed restrictions on using DL in predicting genes of a particular disease. The number of observed genes that caused specific diseases is too small to train a deep learning model. Some algorithms proposed integration between different data sources constructed with multimodal data view [32] using deep learning techniques to identify the valuable features to predict the biological process of genes. Moreover, [33], dgMDL model is proposed to predict associations between all known disease and their genes utilizing DBN rather than predicting only genes of a specific disease. This model effectively increases the number of known genes associated with a specific disease using all known associations. Recently, some researchers applied ensemble learning techniques [34] to effectively predict genes associated with a specific disease. An ensemble learning model is proposed in [35] to improve the classification of heart disease using an ensemble of machine learning techniques collected using a voting strategy to predict disease genes of heart disease. The results show higher accuracy, using ensemble techniques than using a single classifier.

A summary of all recent techniques used in ASD prediction is shown in Table 1, which are machine learning (ML), deep learning (DL), similarity measures (SM), Gene expression (GE), protein-to-protein (PPI) network, gene regulatory network (GRN), and gene ontology (GO). Moreover, the disadvantages of each method showed in Table 2. According to the disadvantages of these methods, we propose a new hybrid ensemble-based classification model,“HEC-ASD,” for predicting ASD genes. The HEC-ASD model utilizes GO to annotate candidates ASD genes and build a functional similarity gene matrix. Moreover, a new hybrid gene similarity (HGS) is proposed to measure the similarity between genes. Different machine learning classifiers are trained and tested in these metrics to evaluate the proposed model. For more improvement, ensemble learning techniques [36] are utilized to enhance the performance of our model, such as Adaptive boosting [37] and Gradient boosting machines [38]. HEC-ASD, based on a Gradient boosting machine, classifies ASD candidate genes effectively with high performance.

**Table 1:** Comparison between ASD research papers using different methods

Studies	ML	DL	Similarity measures		Gene expression	PPI	GRN	GO
			Graph-based	IC-based				
Guan [11]	Yes	No	No	No	Yes	No	No	Yes
Lin [12]	Yes	No	No	No	Yes	No	No	No
Zhang [13]	No	Yes	No	No	No	Yes	No	Yes
Krishnan [14]	Yes	No	No	No	Yes	Yes	Yes	No
Ismail [16]	Yes	No	No	Yes	No	No	No	Yes
Asif [31]	Yes	No	No	Yes	No	No	No	Yes

**Table 2:** Disadvantages of each research method

Studies methods	Disadvantages
ML	In unbalanced dataset, the results will be biased to the majority class
DL	Requires large number of genes to train deep learning model
SM	Long time consuming
GE,PPI,GRN	Neglects some weak interaction, so not be represented in the network
GO	Some genes do not have annotated terms in GO

## Hybrid ensemble-based classification model (HEC-ASD)

**Fig. 1:**
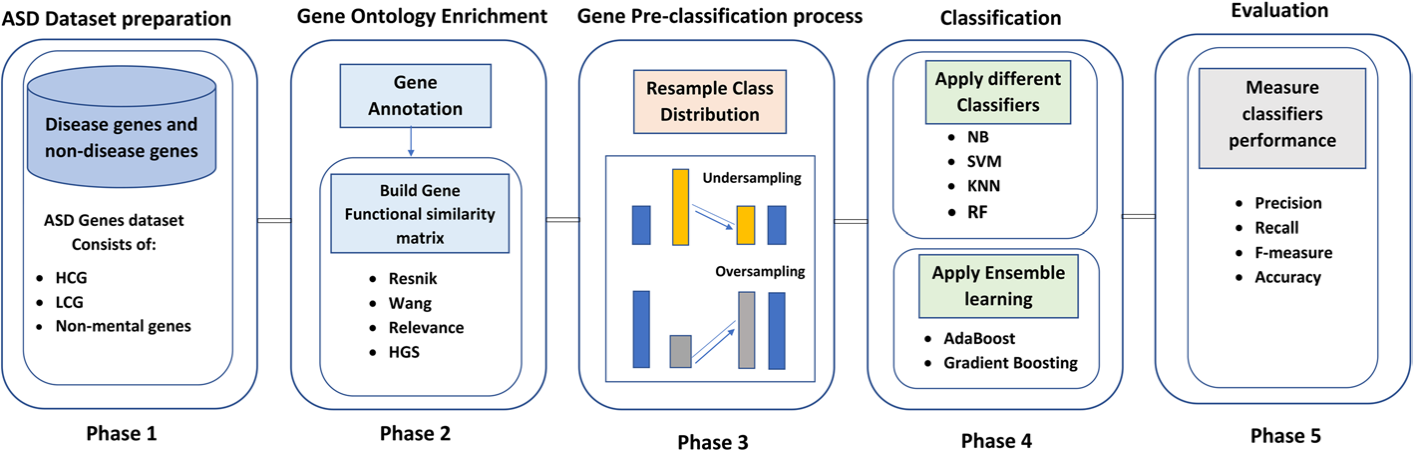
Proposed model (HEC-ASD) framework to predict ASD genes

The general framework of the proposed model is shown in Fig. 1, which consists of five phases: ASD dataset preparation, gene ontology enrichment, Gene pre-classification process, Classification and evaluation. In the first phase, the dataset is collected from the Simons Foundation Autism Research Initiative (SFARI) gene database. SFARI Gene is a database specialized in autism research, which spots gene candidates as one of the autism genes. Secondly, genes are annotated using gene ontology, and the similarity between genes is calculated using different similarity functions such as Resnik, Wang, Relevance, and the proposed hybrid gene similarity (HGS) function. Then, resample the class distribution to be balanced before classification. In the fourth phase, ASD genes are predicted using Random Forest (RF) [39], Support Vector Machine (SVM) [40], Naive Bayes (NB) [41], K-nearest neighbor (KNN) [42], Adaptive boosting (AdaBoost), and Gradient Boosting classifiers to classify ASD genes perfectly. Finally, All classifiers are evaluated using cross-fold validation, and the performance of the classifiers is measured using precision, recall, f-measure, and accuracy.

### ASD dataset preparation

**Fig. 2:**
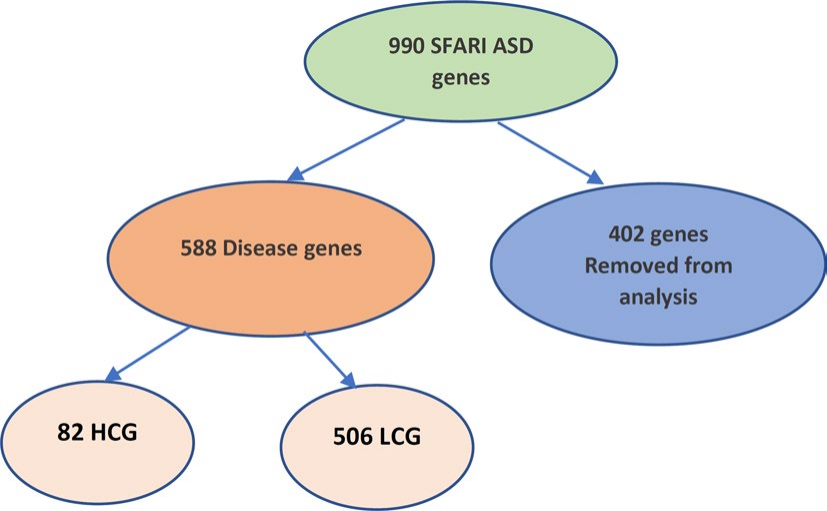
Classification of SFARI database

Simons Foundation Autism Research Initiative (SFARI) gene database https://gene.sfari.org/ is used to assist the proposed model. SFARI contains all genes associated with ASD classified as in Fig. 2. Each gene has an evidence score that reflects how it is associated with the evolution of autism disease. SFARI genes are categorized into seven different categories based on their evidence score. Genes with the highest confidence relating to ASD belong to category one, and genes with less confidence than genes in category one, which may be strong candidates for ASD gene, belong to category two. Categories three and four have the lowest evidence of ASD candidate genes. Category five has an indirect relationship with ASD, and category six is not supported by ASD. Therefore, in this research, categories one, two,three, and four are used for the analysis. Moreover, another type of syndrome gene in a specific column has symptoms or signs which may correlate with ASD. While dataset preparation, only syndrome genes that belong to categories one, two, three, and four will participate in the analysis. SFARI database sets categories one and two as the highest confidence genes (HCG) and three and four as the lowest confidence genes (LCG).

### Gene ontology enrichment

ASD genes are enriched using gene ontology (GO) [15] to calculate the functional similarity between genes. Gene annotation means that each gene is annotated with terms extracted from GO database. The gene ontology (GO) is constructed as a hierarchal graph that annotates genes in terms. Each term in GO is represented with a node, and the relations between nodes are included in the edges. Each term belongs to one of these three categories, which describes the different functions as follows:

- Molecular Function Gene Ontology (MFGO).
- Biological Process Gene Ontology (BPGO).
- Cellular Component Gene Ontology (CCGO).

The gene ontology consists of three core branches. The first one, molecular function, illustrates the activity itself, regardless of the reasons or where these actions could happen. On the other hand, the biological process describes the relation between the initial configurations and the final product, ignoring the mechanism of the process itself. The third is a cellular component that figures the positioning relative to the entire cell structure.

The proposed model focuses on the biological process of gene ontology for analysis. A gene functional similarity matrix must be built to classify the candidate’s ASD genes. Then, measuring the similarity between genes indicates the semantic similarity between their terms. Therefore, if the terms of genes are similar in their semantic value, their genes also must be identical in their functions. Different gene functional similarity methods are used, such as Resnik [43], Relevance [44], and Wang [45]. Resnik and Relevance are information content-based methods (IC), which utilize all the information in the ontology corpus file to measure the semantic similarity between two genes. Wang’s method depends on the structure of GO, so it considers as a graph-based method.

Resnik is based on the information content of terms, which is the negative logarithm of the probability of the term as in Eq. 1.

1 $$\begin{aligned} IC_{t} = - log(Pro(t)) \end{aligned}$$

Pro(t) is the probability of term t, which is the occurrence number of term t in the GO corpus as in Eq. 2. The relationship between IC and the amount of information that this term contains is negative, which means if this term rarely appears in the corpus, it will have more amount of information content.

2 $$\begin{aligned} Pro(t) = \dfrac{\textit{Number Of t}_{\textit{Children}}}{\textit{Total Num of Terms in the Corpus}} \end{aligned}$$

After that, the semantic similarity between the two terms is calculated using the information content of their most common informative ancestor (MICA) as in Eq. 3.

3 $$\begin{aligned} termsimilrity_{Resnik}(t_{1},t_{2}) = IC(MICA) \end{aligned}$$

Relevance method also depends on IC calculations as in Eq. 4

4 $$\begin{aligned} Relevance = \dfrac{2*IC(MICA)(1-Pro(MICA))}{IC(t_{1})+IC(t_{2})} \end{aligned}$$

Wang, in Eq. 5, calculates the similarity between genes terms depending on the position of these terms in the GO-directed graph and their linkage with their ancestors. Therefore, Wang considers the relations is-a and part-of-edges.

5 $$\begin{aligned} similarity_{Wang}(X,Y) = \dfrac{\sum _{t \in T_{X} \cap T_{Y}}S_{X}(t)+ S_{Y}(t)}{SV(X)+SV(Y)} \end{aligned}$$

A hybrid gene similarity (HGS) function is proposed to measure the similarity between two ASD genes. HGS uses Wang as the basic function considering the number of term children, given their ancestor nodes with its descendent nodes. Alg. 1 and 2 illustrate the robust algorithm steps of the HGS method, which helps measure the similarity between two genes. This method uses a GO graph to calculate the number of children nodes rather than using IC values of the term and integrates this number with the Wang method.

**Fig. 3:**
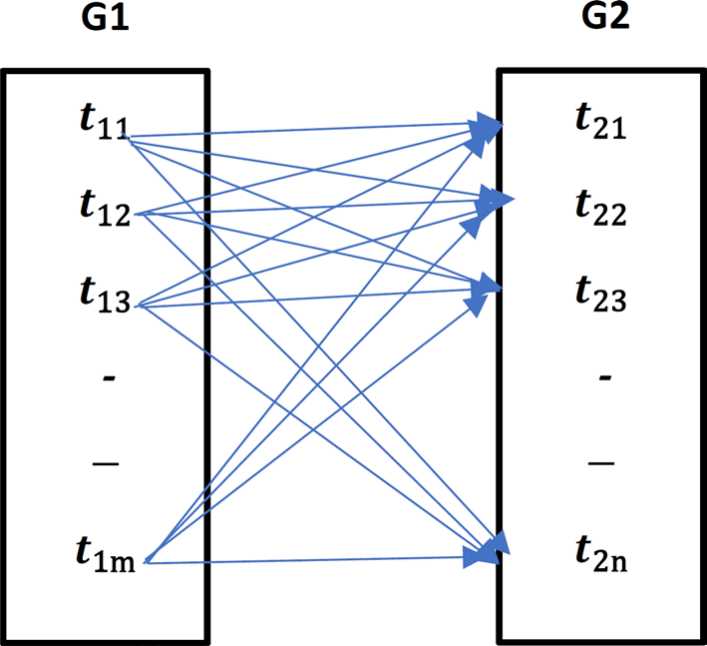
Similarty between two genes annotations

The gene functional similarity matrix should be calculated before gene classification, which is the semantic similarity between genes. Figure 3 represents how we can measure the semantic similarity using their annotated terms from GO. Algo. 1 illustrates the steps to build “TermSimM” which contains all semantic values between two gene terms. Then the average best matching strategy [29] is used to mix the semantic similarity between gene ontology terms. First, we extracted all annotated terms of two genes $g_{1}$ and $g_{2}$. Each term in $g_{1}$ will be calculated with all terms of $g_{2}$ as in Fig. 3. For each term, the directed acyclic graph “DAG” is extracted from GO. DAG of x as in Algo. 1 is the term x with its ancestor terms $T_{x}$ and the edges $E_{x}$ between these terms. GO is represented in three branches (MFGO, BPGO, CCGO). Our experiment involves only BPGO branch. After that, the contributed semantic value of each term is calculated using steps in Algo. 2, which is the semantic function of Wang method using different weight function. The weight $W_{e}$ in Wang method reflect the semantic value of term edges. Researches in [45, 46] find that the number of children of a specific term is negatively related to its IC Value. Therefore, the semantic weight function ($w_{e}$) assigns different values for d constant depending on the type of edge, for part-of relation d equals 0.3 and 0.4 for is-a relationship. The C constant value represents the suitable minimum value of correlation with other methods when c is equal to 0.67. Hence, HGS depends on Wang’s method using the number of the ancestor’s children rather than the information content of ancestor terms. This method saves time when computing the similarity between two genes rather than the IC-based methods.

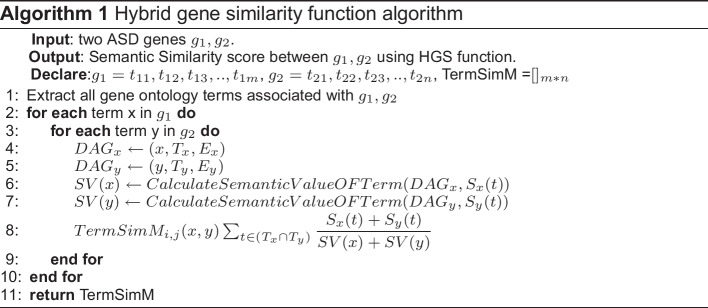

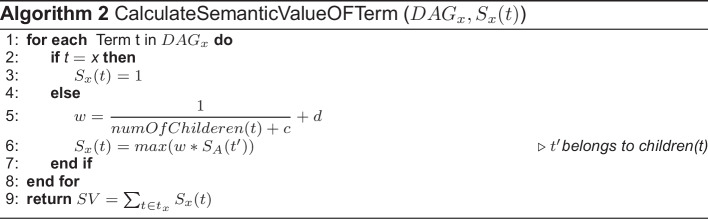





### Gene pre-classification process

The Autism Spectrum database SFARI has a problem of unbalanced class distribution, where the majority class is negative (Not ASD), and the minority class is positive (ASD). Dealing with the dataset as it is will result in false classification with high accuracy, which biases the machine learning classifiers and result in neglecting the minority class. Therefore, dealing with this problem, resample dataset class distribution is the best choice. Resampling techniques can be either deleting some examples randomly from the majority class (random undersampling) or duplicating some examples from the minority class (random oversampling ). To neglect the overfitting of data, random undersampling class distribution skips some of the examples from the majority class randomly until the dataset becomes balanced as in Eq. 6

6 $$\begin{aligned} PrecUnder = \dfrac{\textit{num of positive instances}}{\textit{num of negative intstances}}*100 \end{aligned}$$

### Classification

#### Baseline classifiers

Different machine learning classification techniques are used to evaluate the proposed model, such as Naive Bayes (NB) [41], Support Vector Machine (SVM) [40], K-nearest neighbors (KNN) [42], and Random Forest (RF) [39]. The input for this phase are two functional similarity matrices, one for the highest confidence genes (HCG) and the second for the lowest confidence genes (LCG). Therefore, NB, SVM, KNN, and RF are applied to HCG and LCG. Naive Bayes is a Bayesian classification technique, which is based on calculating the conditional probability that is called the“ Bayes Theorem.” NB method is fast, accurate, and suitable for high dimensional data, but it is considered that all features are independent, which is not acceptable in most applications.

Support Vector Machine is a supervised machine learning technique that treats its predictors as dependent features. SVM draws a separate line to split the input data into groups and then uses this line to predict new data on the place side. SVM seeks to find the most suitable place to put the hyperplane, separating the data into classes, effectively giving high performance. There are two types of SVM, linear SVM and radial SVM. SVM works well with low dimensional data.

#### Ensemble learning techniques

Boosting is one of the ensemble learning techniques utilized to enhance the performance of the proposed model for predicting ASD genes. It is an iterative technique to build a strong learner from a set of weak learners. It corrects the previous model error sequentially, as the second weak learner model attempts to correct the error from the first model, etc. Two different algorithms of boosting are used to propose a more accurate model for predicting autism genes.

- Adaptive Boosting M1 (AdaBoost)
- Gradient Boosting Machines.

**Fig. 4:**
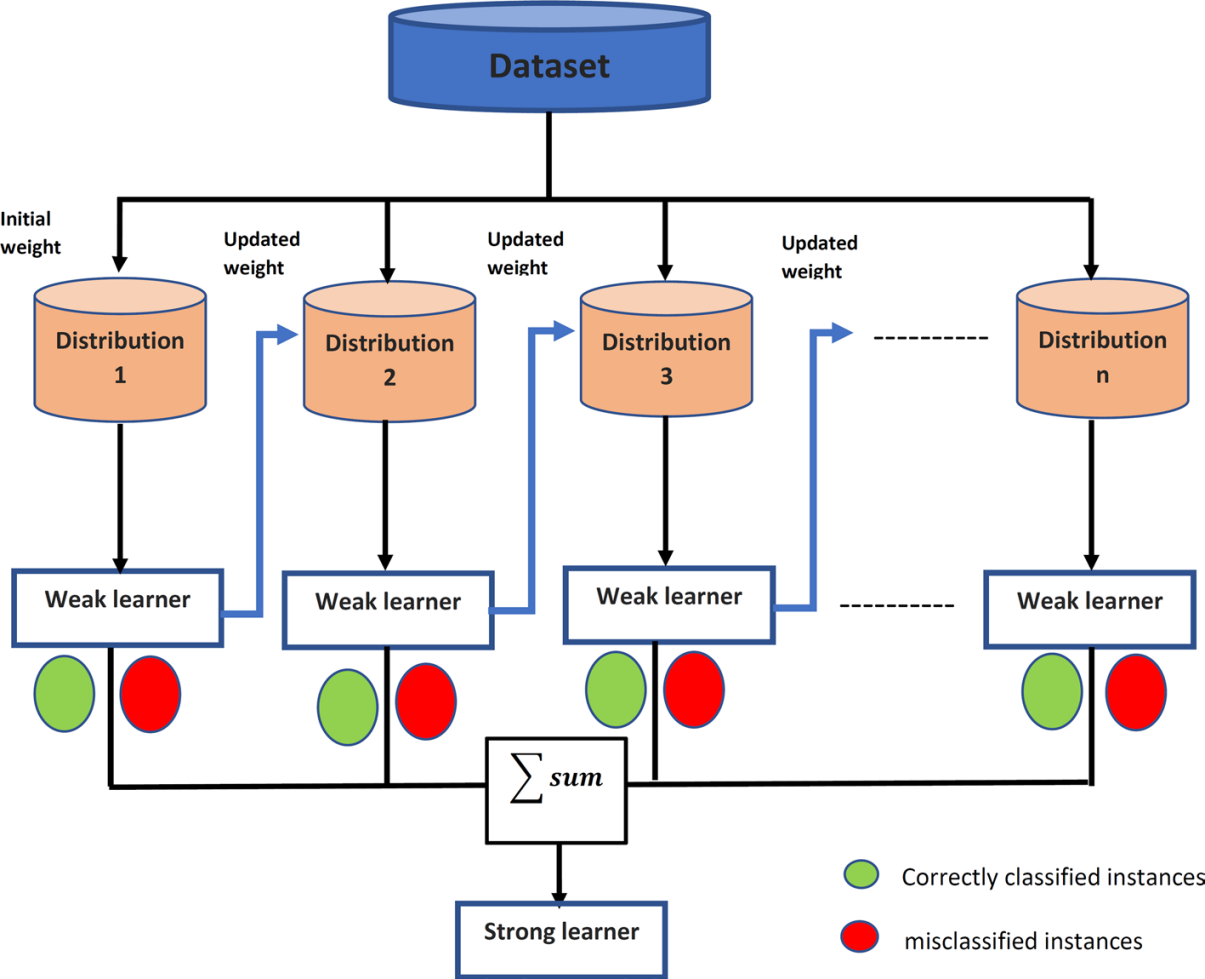
Adaptive boosting general framework

**Adaptive Boosting M1 (AdaBoost)** is the trivial boosting technique, as shown in Fig. 4. It runs at decision stumps as weak learner models, aggregate stronger ones, enhancing the predictive model performance. The steps of the AdaBoost algorithm are in Algo. 3. In the beginning, all training samples are given equal weights, which indicates that all samples are equally important, “one divided by the total number of samples. ”After that, in each iteration of building a new decision stump, these weights will be updated to guide the building of the decision stump (DS). The value of total error and alpha have an opposite relationship; if the total error decreases, then the weak learner (DS) influences the training sample prediction. The total error is a summation of incorrectly classified instance weights. The idea of AdaBoost is to minimize the loss function. In this technique, the exponential loss function gives more weight to misclassified instances and the opposite to correctly classified cases. The algorithm builds decision stumps, either by reaching the number of tree input parameters or the error becomes zero. Finally, the output is a substantial learner prediction value, which is the summation of all hypotheses from the weak learner.

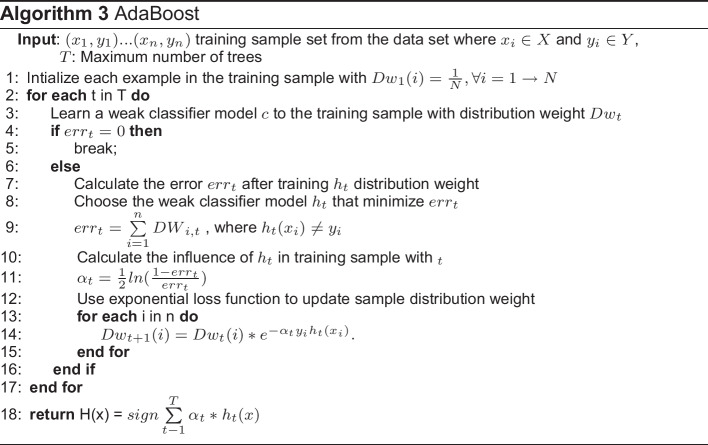



The value of alpha may be positive or negative:

- Positive alpha means that the predicted class label is equal to the actual sample class, which indicates that the samples are correctly classified. Accordingly, the weights for these samples are decreased.
- Negative alpha means that the predicted class label and the actual sample class are unequal, indicating that the samples are not correctly classified. Accordingly, the weights for these samples are increased to build the next weak learners (Decision Stump) to not repeat these misclassified instances in the following stump.

**Fig. 5:**
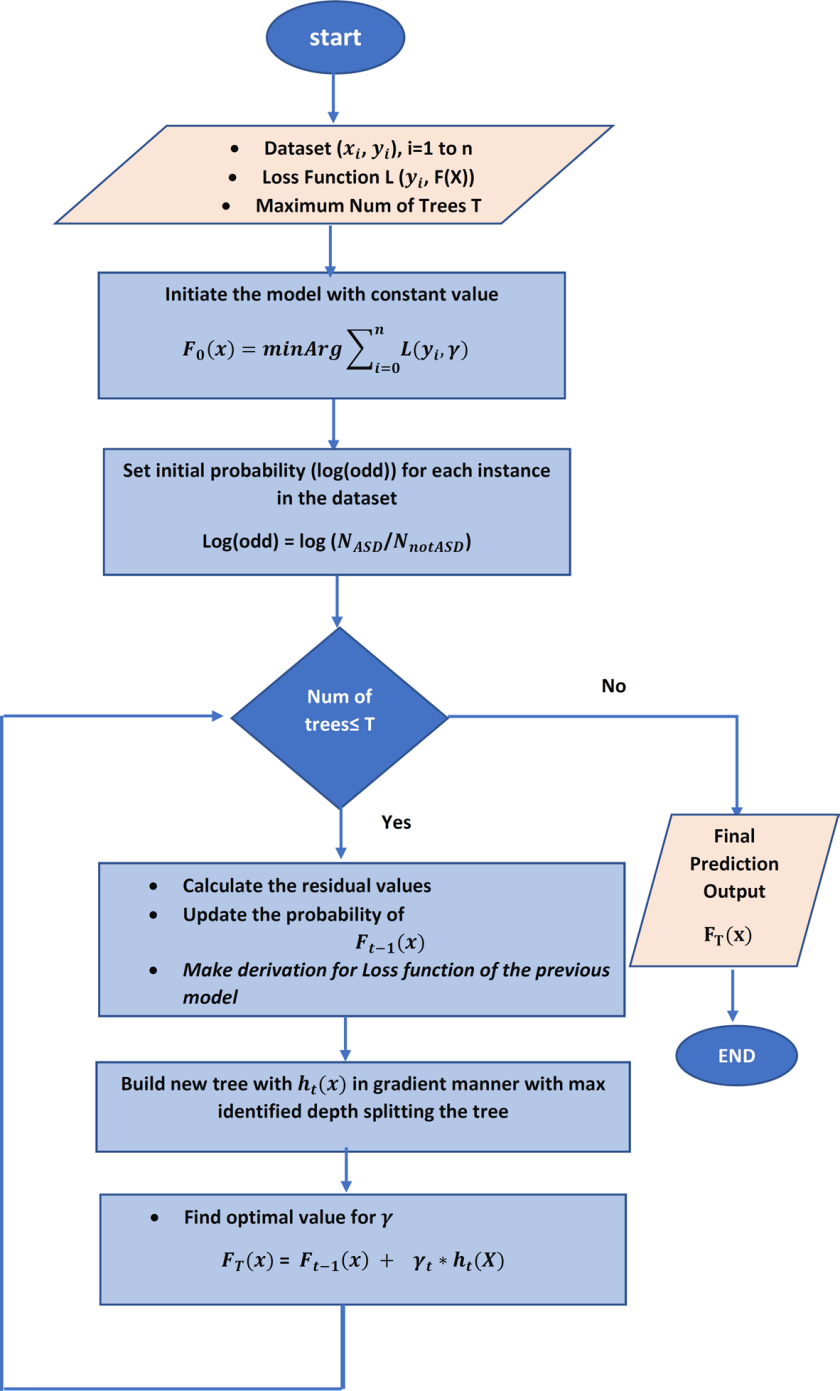
Regularized gradient boosting flowchart

*Gradient Boosting*is another updated boosting algorithm that aims to form strong learners from weak learners using gradient and iterative algorithms. Gradient algorithm proposes to minimize the loss function and must be able to have derivation. Figure 5 shows the main process of the proposed gene prediction-based regularized gradient boosting classification model. HEC-ASD based on gradient boosting depends on four components for enhancing the prediction of ASD genes as follows:

- **Loss Function**, which measures the efficiency of the proposed model in classifying new genes that measure the difference value between the predicted value and the actual observed value.
- **Weak learners** are used in the training phase, which results in low accuracy with high error; decision stumps are utilized to be the weak learner.
- **Additive model**, which means that the model works sequentially, adding trees (weak learners) iteratively and additive. In each iteration, the loss function should be decreased to form a stronger learner model.
- **Regularization parameters** are parameters used to regulate the loss function to prevent overfitting or underfitting problems. The parameters are the number of trees, learning rate, maximum depth, and lambda l2 regularization. The learning rate is used to decrease the iterative gradient steps. Lambda ? l2 regularization is a hyperparameter that measures the regulation degree.

HEC-ASD, based on gradient boosting, utilized the Log loss function to minimize the total prediction error using Eq. 7, where $y_{i}$ is the actual observed class value.

7 $$\begin{aligned} log loss = -\frac{1}{N}\sum \limits _{i=1}^{N}y_{i}*log(p(y_{i}))+(1-y_{i})*log(1-p(y_{i})) \end{aligned}$$

## Experimental results

### Database

The proposed prediction model uses SFARI database for performance assessment. SFARI database sets categories one and two as the highest confidence genes (HCG), and categories three and four as the lowest confidence genes (LCG). SFARI gene database contains 990 genes associated with ASD, 82 genes from them classified as HCG, which are the genes with the highest evidence, 506 genes classified as LCG, which are the genes with the lowest evidence associated with ASD. The rest 402 genes are excluded from the analysis in the case of syndrome genes not belonging to any category from 1 to 4, also with genes that have no evidence score or have hypothesis evidence. Moreover, 1189 genes annotated as non-mental genes are included in the analysis process, which is considered as negative examples (non-ASD genes). A random undersampling is used to solve the showed imbalance in class distribution.

### HEC-ASD evaluation

The proposed model for predicting ASD genes is evaluated using a stratified cross-fold validation assessment till five-fold. This assessment does the following steps in validating data:

- Split the dataset into equal five folds.
- Use four folds as training data.
- Use the remaining fold as testing data.
- These steps are repeated five times and chosen in the diverse fold for testing

### Performance measures

Four different performance metrics are used to measure the performance of the proposed classification method, recall in Eq. 8 , precision in Eq. 9, f-measure in Eq. 10, and accuracy in Eq. 11. The term True Positive (TP) refers to the number of the documents correctly selected to this class. True Negative (TN) is the number of documents correctly rejected to be chosen for this class label. False Positive (FP) is the number of documents incorrectly rejected that was selected for this class label. False Negative (FN) is the set of documents incorrectly chosen for this class label.

8 $$\begin{aligned} Recall = \dfrac{TP}{TP + FN} \end{aligned}$$

9 $$\begin{aligned} Precision = \dfrac{TP}{TP + FP}\end{aligned}$$

10 $$\begin{aligned} F\text {-}measure =\dfrac{2* Precision * Recall}{Precision + Recall}\end{aligned}$$

11 $$\begin{aligned} Accuracy = \dfrac{TP+TN}{TP+FP+TN+FN} \end{aligned}$$

Two versions of the gene functional similarity matrix are constructed, one using the highest confidence genes (HCG) and non-mental genes from Krishnan et al., and the second for both highest and lowest confidence genes, and non-mental genes (HCG+LCG+non-mental genes). These versions of data are built using Wang, Relevance, and Resnik semantic similarity measures and then tested using different basic classifiers in the first step, such as Naive Bayes (NB), Random Forest (RF), Support vector machine (SVM), and K-nearest neighbors (KNN). Table 3 illustrates a comparison of these two versions of matrices in terms of accuracy. The data version of (HCG+non-mental) showed the highest performance compared to the other version of the data especially using Random Forest classifier with a Resnik similarity measure 80%.

**Table 3:** The performance of different classifiers evaluated on HCG and LCG SFARI dataset using different semantic similarity measures

SM measure	Classifier	HCG+non-mental %	HCG+LCG+non-mental %
Resnik	NB	71.8	66.7
	**RF**	**80**	75.9
	SVM	48.6	59.6
	KNN	78.4	67.3
Relevance	NB	70.6	65
	RF	76.8	74.8
	SVM	52.4	61
	KNN	74.9	63
Wang	NB	71.5	62.7
	RF	74.5	74
	SVM	54	58.6
	KNN	70.3	60.6

**Fig. 6:**
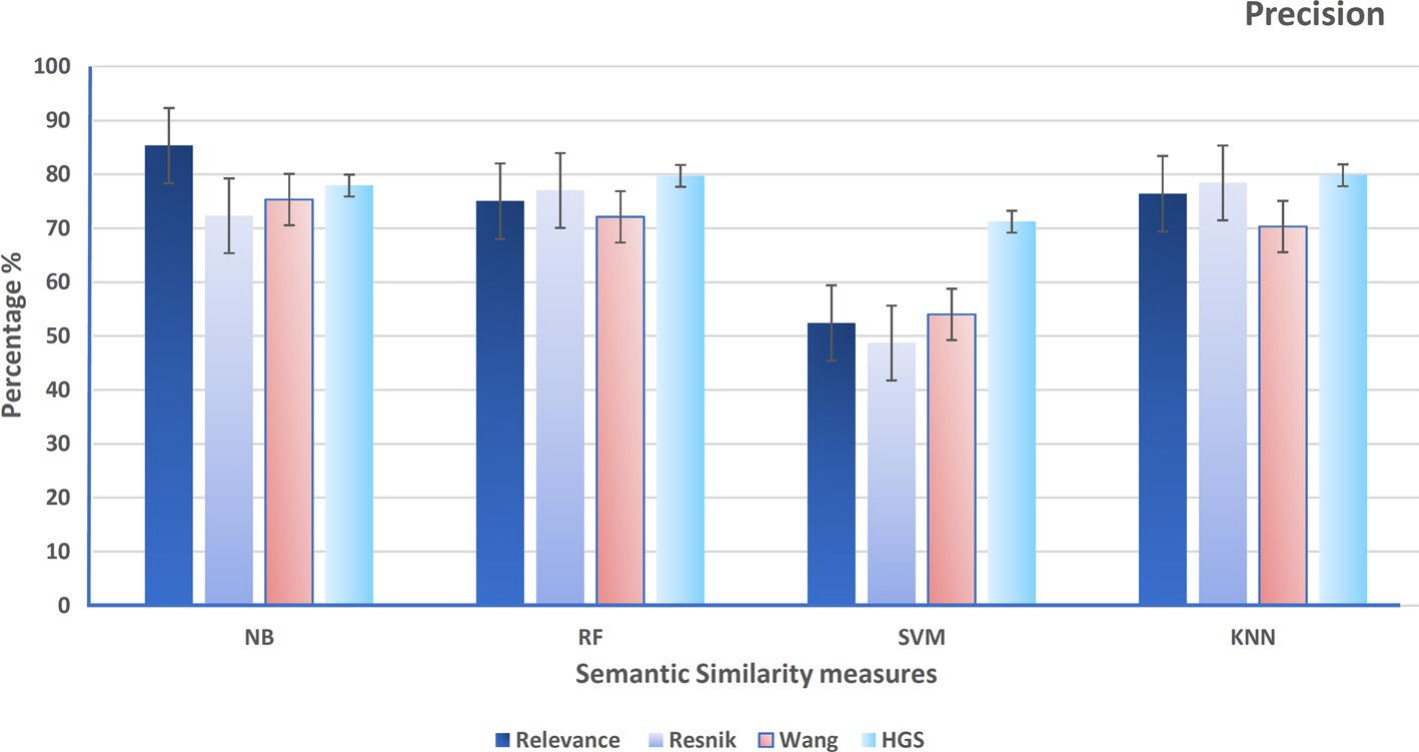
The performance of different classifiers evaluated on SFARI dataset using different semantic similarity measures in terms of precision

**Fig. 7:**
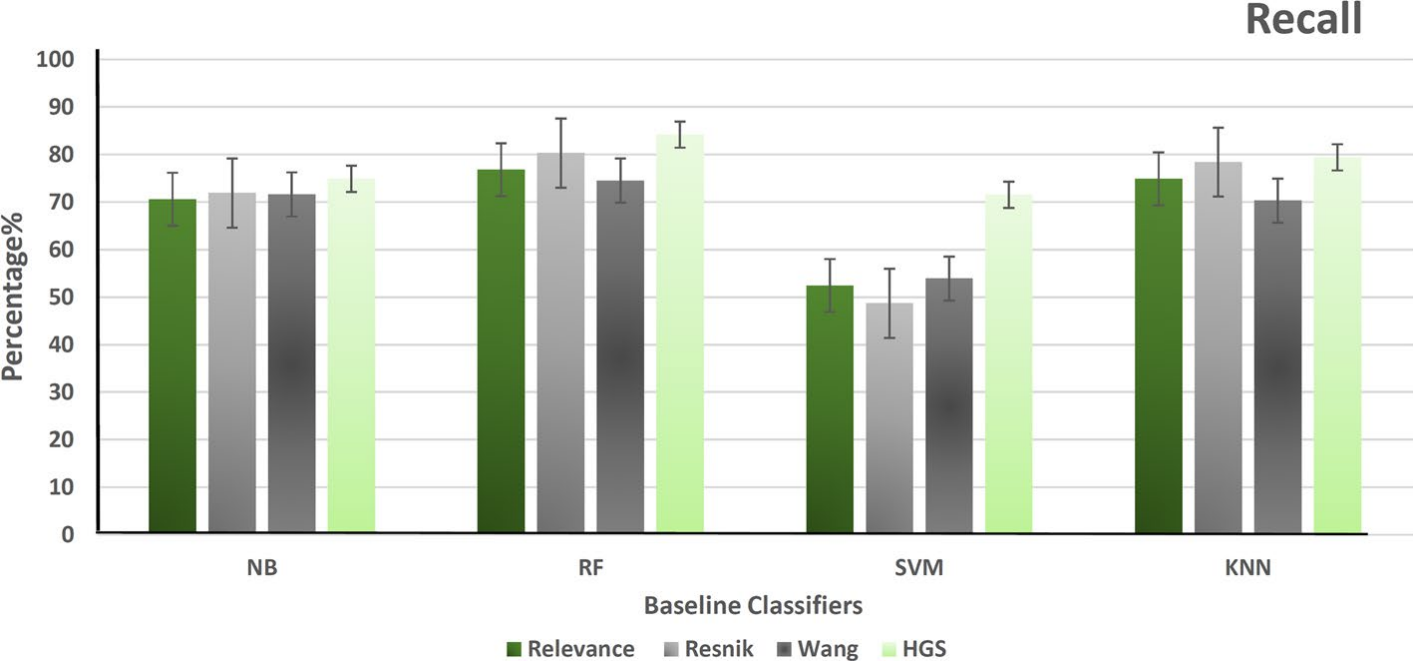
The performance of different classifiers evaluated on SFARI dataset using different semantic similarity measures in terms of recall

**Fig. 8:**
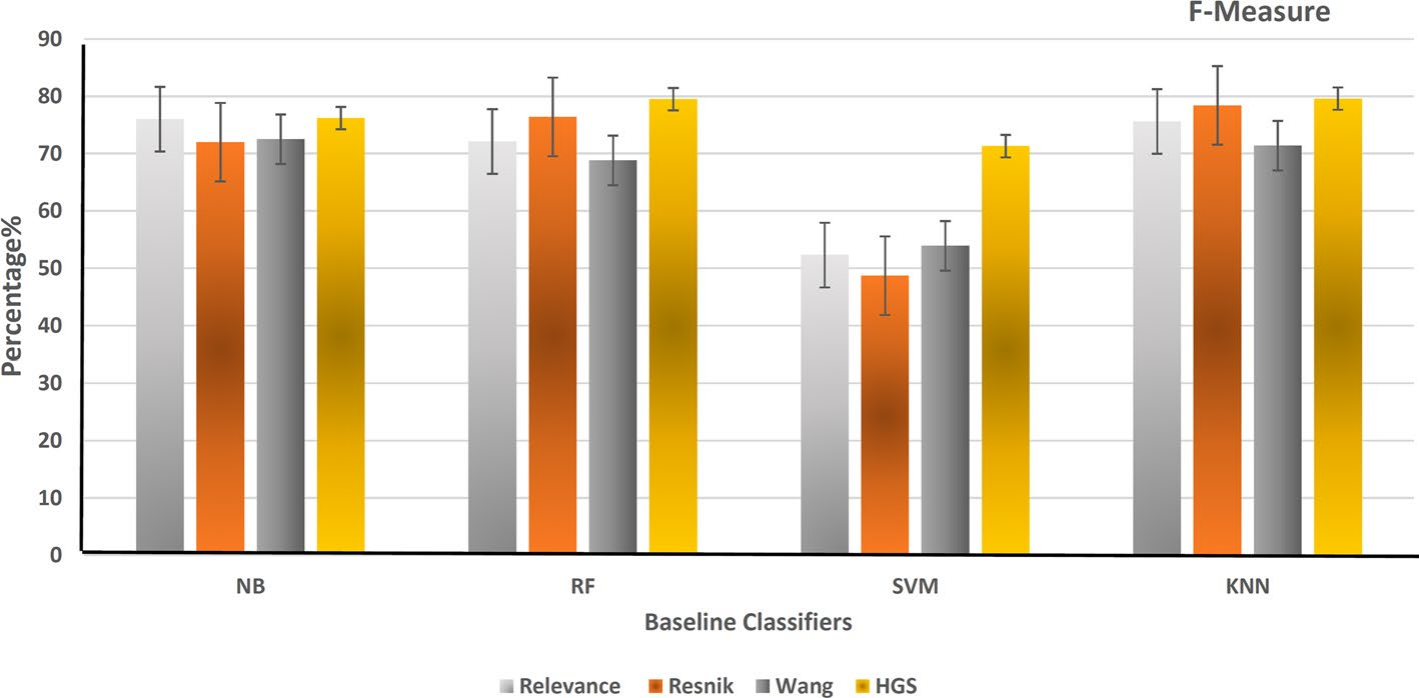
The performance of different classifiers evaluated on SFARI dataset using different semantic similarity measures in terms of f-measure

**Fig. 9:**
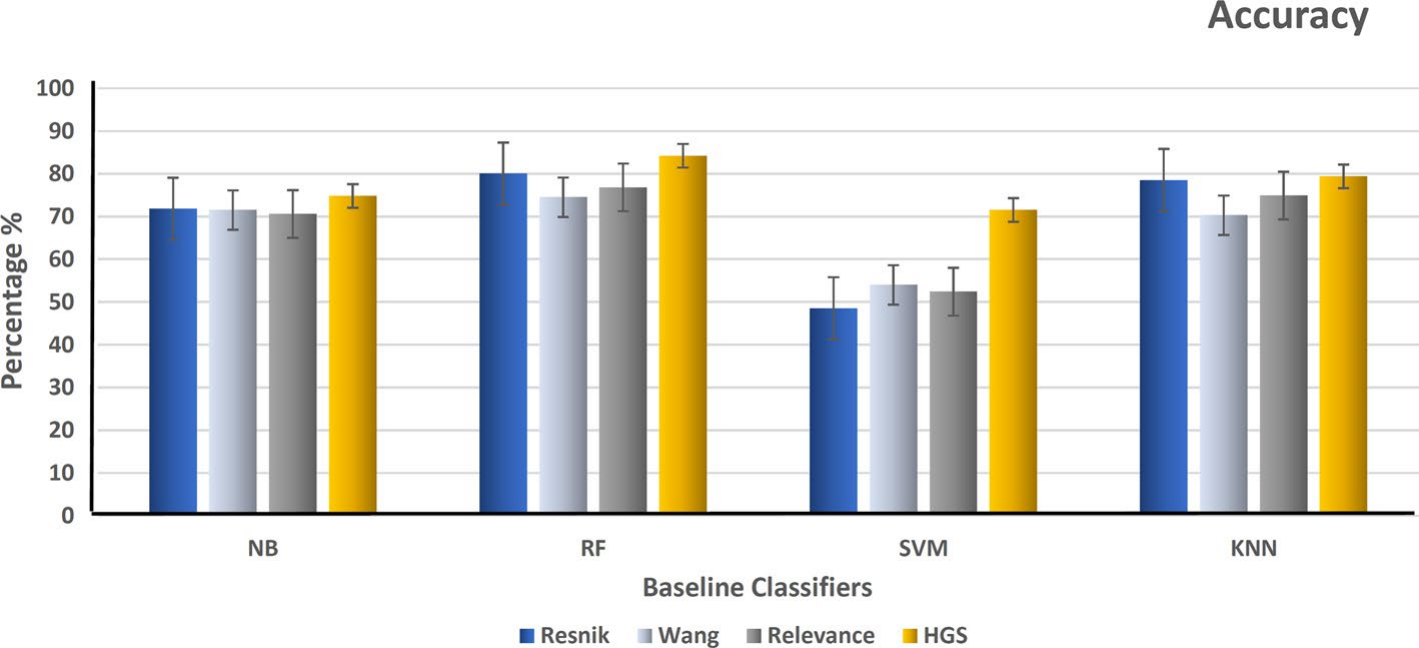
The performance of different classifiers evaluated on SFARI dataset using different semantic similarity measures in terms of accuracy

**Fig. 10:**
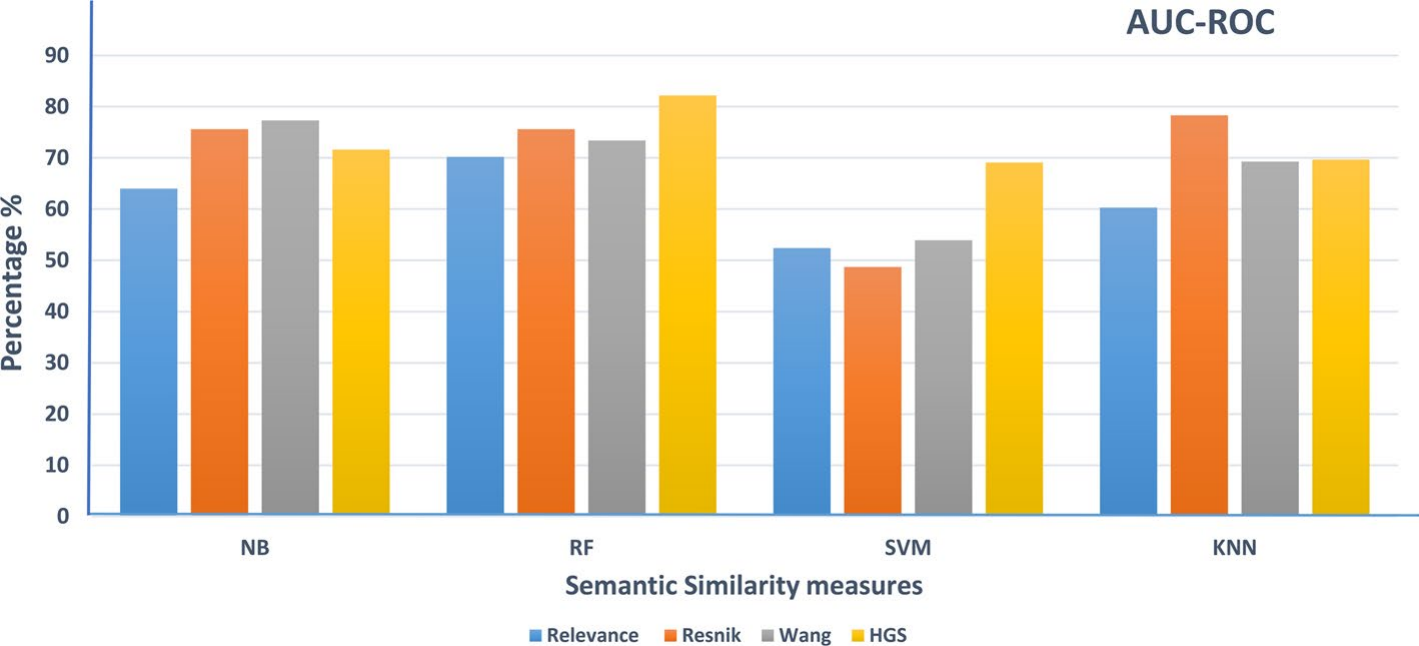
The performance of different classifiers evaluated on SFARI dataset using different semantic similarity measures in terms of AUC-ROC

After that, hybrid gene similarity (HGS) is applied to the version of the data (HCG+ non-mental genes). The four functional similarity measures (Wang, Resnik, Relevance, and HGS) are used to represent the results in terms of precision, recall, F-measure, accuracy, and the area under the curve of the receiver operating characteristic (AUC-ROC). Figure 6 shows the precision of different classifiers using the four similarity measures that represent the ratio of positive gene samples that are correctly classified. Figure 7 represents the ratio of real positive genes predicted correctly in terms of recall. Moreover, Figs. 8, 9, and 10 show the proposed model’s results in F-measure, accuracy, and AUC-ROC. The HGS method reached an improved accuracy of 84% using a Random Forest classifier compared with the highest reached accuracy using a Resnik method with Random Forest, which reached 80%. This improvement using the hybrid HGS method indicates a valuable measure in enhancing the prediction of new ASD genes.

**Fig. 11:**
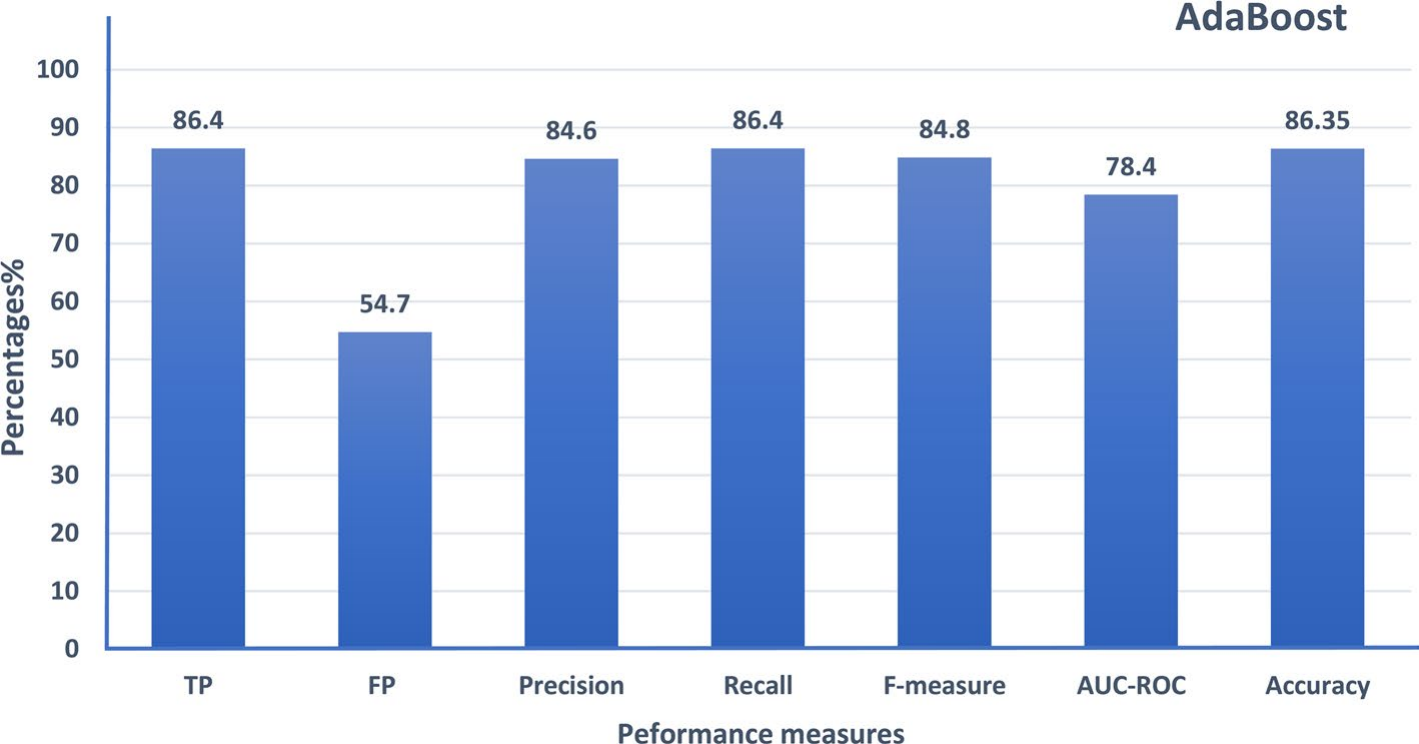
The performance of AdaBoost ensemble boosting classifier evaluated on SFARI dataset using HGS semantic similarity measure

Ensemble learning techniques are utilized in the proposed model to enhance its performance. The first part used the hybrid gene similarity function (HGS) with AdaBoost ensemble learning machines. Figure 11 shows a detailed performance measure in terms of true positive rate (TP Rate), false positive rate (FP Rate), Precision, Recall, F-measure, AUC-ROC and Accuracy, which reached 84.35%, increasing the accuracy of Random Forest by around 4.5%.

Moreover, HEC-ASD based on a gradient boosting model used regularization parameters to prevent overfitting the model. The model is tested using num of trees = 500, learning rate = 0.1, limited depth of individual trees = 3, and lambda= 5 (regularization parameter). The results are shown using the area under the curve of ROC (AUC-ROC), f-measure, precision, recall, classification accuracy, and specificity performance measure. Specificity in Eq. 12, indicates that high specificity refers to a lower error rate.

12 $$\begin{aligned} specificity = \dfrac{\textit{number of TN}}{\textit{number of TN + number of FP}} \end{aligned}$$

**Fig. 12:**
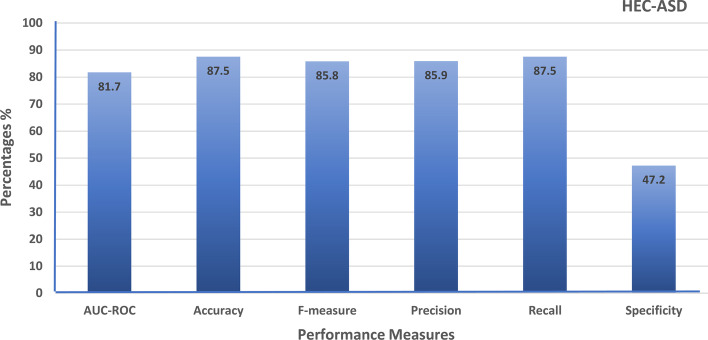
The performance of HEC-ASD based on gradient boosting classifier evaluated on SFARI dataset using HGS semantic similarity measure

Figure 12 contains the results of the proposed HEC-ASD based on a gradient boosting model, the model getting the highest accuracy with a low error rate as gets the highest Specificity. The proposed HEC-ASD model gained promising results of around 88% compared to Krishnan et al. [14] and [16, 31], which gained 73% and a maximum 80% in [16]. Krishnan et al. use a gene regulatory network and protein-to-protein network to predict the ASD genes, and in [16] gained improved performance than Krishnan et al. using the basic classifiers using the basic semantic measures. HEC-ASD outperforms both methods using gene ontology as a reference database for genes utilizing new hybrid gene similarity function (HGS), which improves the accuracy of the proposed model to 84%. Ensemble boosting techniques using AdaBoost gained enhancement to 86% and gradient boosting is used to propose the highest-performed model HEC-ASD which increase the performance of the proposed model to around 88%.

## Discussion and interpretation

Autism spectrum disorder (ASD) is a complex disease diagnosed mainly from the outward symptoms and behavior of the child. Moreover, ASD lacks genetic causes. In this study, the HEC-ASD model is proposed to predict genes related to ASD. HEC-ASD model outperforms the reported model in [14] that used a weighted SVM model utilizing the information in gene expression, gene regulatory, and PPI network. The reported model [14] is selected to be the basic state-of-art method to compare with our proposed model. The comparison is conducted as a HEC-ASD model uses the same dataset and machine learning techniques using the same measurement. The proposed HEC-ASD model makes the same processes in [14] with different behavior. HEC-ASD enrichment technique utilizes gene ontology (GO) to annotate genes with terms. Moreover, the proposed HEC-ASD model used different semantic similarity functions to construct a gene functional similarity matrix. The results of HEC-ASD showed that the Random forest classifier using Resnik showed the highest accuracy compared to other basic classifiers such as NB, SVM, and KNN. More improvement using ensemble learning techniques such as AdaBoost and gradient boosting machines. Moreover, a hybrid gene similarity function (HGS) is proposed to measure the semantic similarity between genes. The proposed model using gradient boosting with HGS outperforms other classifiers, resulting in a performance of around 88 %. The proposed HEC-ASD model is trained and tested on HCG and non-mental genes and outperforms the reported model [14], which reaches an accuracy of 73%.

The improvement in the classifiers’ performance using the HGS function approved that HGS semantic similarity is an effective method to measure similarities between genes as follows:

- HGS takes the benefits from information content and the Wang method, so it is a hybrid method.
- HGS is fast, as there is no need to count the IC for gene terms from a large corpus GO.
- HGS considers the number of child nodes for gene terms instead of their IC values in calculating the semantic similarity between the terms.

Moreover, it confirmed the importance of using GO rather than PPI, gene expression, and gene regulatory network reported in [14], which neglects some interaction between genes. Furthermore, ensemble learning techniques improve the performance of the proposed predictive model, minimizing model errors. The limitation in our proposed model is that certain genes are not included in the analysis process because these genes have not annotated GO terms. This limitation does not affect the performance because their number is low compared to the number of genes. In the future, more improvements can be made integrating some other annotation resources with GO; also, GO is usually updated with more information, which may solve this limitation.

## Conclusion

Complex diseases such as autism lack the presence of genetic causes of the disease, as most research has focused on the environmental causes of the disease. Therefore, identifying disease-causing genes is a challenge. An effective methodology for predicting genes cause ASD is proposed using gene ontology (GO) to overcome the problems using protein to protein interactions (PPI) network, which is used in state-of-the-art methods. Using GO to calculate genes’ functional similarities, which enhances predicting ASD genes. The semantic similarity between genes is between zero and one score using different similarity measures. A new hybrid semantic similarity function was used, which is a hybrid between information content methods and Wang method. This measure showed improved accuracy than traditional semantic measures evaluated using different classifiers. Random Forest (RF) classifier evaluated on high confidence genes using a hybrid gene similarity function (HGS) showed better performance than others classifiers. Moreover, a hybrid ensemble-based classification model (HEC-ASD) using regularized gradient boosting is proposed. HEC-ASD is boosting techniques, building models iteratively and sequentially, where each model seeks to correct the previous model errors. HEC-ASD gets the highest improvement in accuracy predicting ASD disease genes compared with other models that used protein to protein networks and gene expression or gene regulatory networks. The results obtained from HEC-ASD model get the highest performance accuracy, 88%, compared with other techniques, which gained 73%. This effective improvement indicates that gene ontology is effective in annotating genes, as it contains updated information about genes, and using gradient ensemble learning machines helps get an efficient model for predicting ASD disease genes automatically.

## Data Availability

The Simons Foundation Autism Research Initiative (SFARI) gene database, which analyzed during the current study, is available at https://gene.sfari.org/.
